# *Neisseria gonorrhoeae* Strain with Reduced Susceptibilities to Extended-Spectrum Cephalosporins

**DOI:** 10.3201/eid2007.131396

**Published:** 2014-07

**Authors:** Duylinh Nguyen, Severin Gose, Lina Castro, Kathleen Chung, Kyle Bernstein, Micheal Samuel, Heidi Bauer, Mark Pandori

**Affiliations:** San Francisco Department of Public Health, San Francisco, California, USA (D. Nguyen, S. Gose, L. Castro, K. Chung, K. Bernstein, M. Pandori);; California Department of Public Health, Richmond, California, USA (M. Samuel, H. Bauer)

**Keywords:** Neisseria gonorrhoeae, bacteria, antimicrobial resistance, reduced susceptibilities, extended-spectrum cephalosporins, cephalosporins, strain, ST1407, genogroups, California, United States

## Abstract

The spread of *Neisseria gonorrhoeae* strains with reduced susceptibility to extended-spectrum cephalosporins is an increasing public health threat. Using Etest and multiantigen sequence typing, we detected sequence type 1407, which is associated with reduced susceptibilities to extended-spectrum cephalosporins, in 4 major populated regions in California, USA, in 2012.

*Neisseria gonorrhoeae* infections are the second most common sexually transmitted infection in the United States (*1*). Gonorrhea typically presents as urethritis in men and cervicitis in women but when left untreated can result in severe sequelae, such as infertility (*2*,*3*). Gonorrhea infections continue to be a public health problem worldwide, and control efforts have been complicated because of the ability of the organism to develop resistance to all first-line antimicrobial drugs used in treatment, including penicillins, tetracyclines, and fluoroquinolones.

Treatment failures and isolates with reduced susceptibilities to extended-spectrum cephalosporins (ESCs) have been detected in Asia (*4*,*5*), Canada (*6*), Europe (*7*,*8*), and South Africa (*9*). In the United States, ESCs are currently the foundation of treatment recommendations. However, the increasing prevalence of isolates with reduced susceptibility to ESCs has led to dual treatment with ceftriaxone plus azithromycin or doxycycline. This combination is the only recommended treatment regimen of the US Centers for Disease Control and Prevention (CDC) (*10*).

*N*. *gonorrhoeae* isolates with reduced susceptibility to ESCs have been linked to altered penicillin-binding protein, which is encoded by the *penA* gene. Molecular and genomic epidemiologic studies have been used to describe *N*. *gonorrhoeae* antimicrobial drug resistance to ESCs in much of the world. In the United States, isolates with reduced susceptibility to cefixime have been associated with a specific strain of *N. gonorrhoeae* (*11*). However, little is known about the molecular epidemiology of strains with reduced susceptibilities to ceftriaxone, the current recommended ESC. In this study, we conducted surveillance for genotypic and phenotypic diversity of *N*. *gonorrhoeae* strain diversity in California during January 2012–September 2013.

## The Study

Urethral gonococcal isolates were obtained from men with urethral infections during the CDC-sponsored Gonococcal Isolate Surveillance Project (GISP) at sites in Los Angeles, Orange, San Diego and San Francisco Counties in California. Duplicates of all GISP isolates from these sites were analyzed at the San Francisco Department of Public Health Laboratory by using Etest and at the GISP regional laboratory by using agar dilution. Antimicrobial drug susceptibility testing was performed by using Etest (bioMérieux, Marcy l’Etoile, France) as part of the California rapid response project to enhance surveillance efforts aimed at detecting treatment failures and containing the spread of isolates that require increased MICs of third-generation cephalosporins.

A total of 718 isolates were tested for susceptibility to ceftriaxone, cefixime, and azithromycin by Etest. In brief, frozen isolates were thawed in a biosafety cabinet at room temperature, inoculated onto a chocolate II agar plate containing 1% Iso VitaleX (Becton Dickinson, Franklin Lakes, NJ, USA), and incubated at 37°C with in an atmosphere of 5% CO_2_ for 20–25 h. A sterile swab was then used to collect colonies on the plate and suspend them in typticase soy broth containing 15% glycerol to give a turbidity of 0.5–1 MacFarland units. Using another sterile swab, we plated out the liquid culture onto 3 chocolate II agar plates in three 90° turnings. We then placed an Etest strip in the center of each plate by using a manual applicator. After incubation for 20–25 h at 37°C in an atmosphere of 5% CO_2_, the plates were examined, and MICs of each drug were recorded according to the manufacturer’s instructions.

Molecular epidemiologic typing was performed by using *N*. *gonorrhoeae* multiantigen sequence typing (NG-MAST) on all isolates. NG-MAST was performed as described (*12*) and allele numbers of *porB* and *tbpB* genes and sequence types (STs) were assigned by using the NG-MAST website (http://www.ng-mast.net).

Of 718 urethral isolates that were sequence typed, 258 STs were identified that had 192 *porB* alleles and 60 *tbpB* alleles. Eighty-three STs were present among ≥2 isolates in the collection, and the remaining 175 STs were unique. The 5 most prevalent STs identified were ST2400 (55, 7.7%), ST2992 (46, 6.4%), ST3307 (45, 6.3%), ST1407 (38, 5.3%), and ST7268 25, 3.5%). These STs constituted 29% of all isolates obtained. ST1407, the fourth most prevalent ST in this study, has been associated with ESC treatment failures in Europe and Canada. In California, this ST and closely related STs were associated with reduced susceptibility to ESCs, but these STs remained susceptible to azithromycin.

Because of the high discriminatory power of NG-MAST, which assigns STs on the basis of single basepair differences, we sought to assess diversity on the basis of genogroups. Genogroups were defined as having identical *tbpB* alleles and ≥99% DNA sequence similarity within *porB* alleles as defined by NG-MAST (*13*). The 718 urethral isolates sequence typed for this study formed 56 genogroups (≥2 isolates) and 92 ungrouped isolates with unique STs. The 5 most common genogroups were G2992 (n = 70, 9.7%), G2400 (n = 59, 8.2%), G3307 (n = 52, 7.2%), G1407 (n = 45, 6.3%) and G3935 (n = 31, 4.3%) (Table 1, Appendix).

**Table 1:** Five most prevalent genogroups for *Neisseria gonorrhoeae* isolates from symptomatic male patients in 4 counties in California, USA, 2012

County	Total no. isolates	Genogroup, no. (%) isolates				
		G2992	G2400	G3307	G1407	G3935
San Francisco	246	32 (13)	29 (11.7)	13 (5.3)	12 (4.9)	9 (3.6)
Los Angeles	147	6 (4)	13 (8.8)	21 (14.3)	15 (10.2)	13 (8.8)
Orange	121	14 (11.6)	6 (5)	8 (6.6)	5 (4)	6 (5)
San Diego	204	18 (8.8)	15 (7.4)	12 (5.9)	19 (9.3)	4 (2)
Total	718	70 (9.7)	59 (8.2)	52 (7.2)	45 (6.3)	31 (4.3)

Most isolates in our study required low MICs (≤0.023 μg/mL) for ceftriaxone and cefixime. Proportions of low, intermediate, and high MICs required by isolates in the 5 most prevalent genogroups are shown in Table 2. The G2400 and G1407 isolates were the only genogroups to require predominantly intermediate MICs of ceftriaxone and cefixime (0.032−0.094 μg/mL). G1407 comprised 91% (10/11) of isolates that required a high MIC of ceftriaxone and 93% (14/15) of isolates that required a high MIC of cefixime. Overall, 99.7% (716/718) of isolates required an MIC of azithromycin ≤1.5 μg/mL, of which 88% (629/716) required an MIC of azithromycin ≤0.380 μg/mL. Only 2 isolates required an MIC of azithromycin ≥2.0 μg/mL (2.0 μg/mL and 3.0 μ /mL).

**Table 2:** MICs required for *Neisseria gonorrhoeae* isolates of the 5 most common genogroups in 4 counties in California, USA, 2012*

Table 2. MICs required for *Neisseria gonorrhoeae *isolates of the 5 most common genogroups in 4 counties in California, USA, 2012*											
Genogroup	Cefixime, no. (%)				Ceftriaxone, no. (%)				Azithromycin, no. (%)		
	Low	Intermediate	High		Low	Intermediate	High		Low	Intermediate	High
2992	69 (98.5)	1 (0.015)	0		70 (100)	0	0		52 (74.3)	18 (25.7)	0
2400	19 (32)	40 (68)	0		8 (13.6)	51 (86.4)	0		55 (93)	4 (7)	0
3307	51 (98)	1 (2)	0		51 (98)	1 (2)	0		50 (96)	2 (4)	0
1407	4 (9)	27 (60)	14 (31)		7 (15.5)	28 (62.2)	10 (22.3)		26 (58)	19 (42)	0
3935	30 (97)	1 (3)	0		30 (97)	1 (3)	0		14 (45)	17 (55)	0
Other	436 (94.5)	24 (5)	1 (0.5)		384 (83.3)	76 (16.5)	1 (0.02)		432 (94)	27 (5.8)	2 (0.2)

*Cefixime: low ≤0.023 μg/mL, intermediate 0.032–0.094 μg/mL, high ≥0.125 μg/mL; ceftriaxone: low ≤0.023 μg/mL, intermediate 0.032–0.094 μg/mL, high ≥0.125 μg/mL; azithromycin: low ≤0.380 μg/mL, intermediate 0.5–1.5 μg/mL, high ≥2.0 μg/mL.

Seventeen isolates that had reduced susceptibility phenotypes for ESCs (ceftriaxone MIC ≥0.125 μg/mL and cefixime MIC ≥0.125 μ /mL) were subjected to *penA* sequencing to determine the presence an altered *penA* gene. Sequencing was performed as described (*14*). Sequencing of *penA* genes showed that 100% (17/17) had the mosaic *penA* XXXIV allele. Genetic characteristics of isolates that required high MICs of ESCs are shown in Table 3. The *tbpB* 110 allele was found in 100% (17/17) of the isolates that required high MICs of ESCs Cs, and 94% (16/17) were G1407.

**Table 3:** Genetic characteristics of, and MICs required for, 17 *Neisseria gonorrhoeae* isolates of the 5 most common genogroups in 4 counties, California, USA, 2012*

Isolate	Genogroup	ST	*porB* allele	Ceftriaxone MIC	Cefixime MIC	Azithromycin MIC
1	1407	1407	908	0.25	0.25	0.75
2	1407	1407	908	0.25	0.19	0.75
3	1407	1407	908	0.25	0.19	0.5
4	1407	1407	908	0.19	0.125	1.0
5	1407	1407	908	0.125	0.125	0.38
6	1407	1407	908	0.125	0.125	0.25
7	1407	1407	908	0.125	0.125	0.75
8	1407	1407	908	0.125	0.125	0.5
9	1407	1407	908	0.094	0.125	0.38
10	1407	1407	908	0.094	0.125	0.5
11	1407	1407	908	0.064	0.125	0.38
12	1407	1407	908	0.047	0.125	0.38
13	1407	1407	908	0.125	0.094	0.75
14	1407	1407	908	0.125	0.094	0.75
15	1407	3158	1914	0.094	0.125	0.19
16	1407	8417	1903	0.094	0.125	0.38
17	Unique	8476	4878	0.125	0.125	0.5

*All 17 isolates had the 110 allele for *tbpB* and the XXXIV allele for *penA*. MICs are shown in micrograms per milliliter. ST, sequence type.

## Conclusions

Genotypic surveillance of *N*. *gonorrhoeae* has been performed in the United States and elsewhere, but little is known about the genetic diversity of strains circulating in the United States, according to CDC recommended treatment guidelines. We sought to perform phenotypic and genotypic surveillance of isolates from major population centers in California in 2012. ST1407, a widespread strain linked to ESC treatment failures, was found in all 4 counties in California and was the fourth most prevalent ST overall.

In this study, ST1407 was associated with reduced susceptibility to ceftriaxone and cefixime. These data show that ST1407 is well established in California and are consistent with results from Asia (*4*,*5*), Canada (*15*), Europe (*8*), and the United States (*11*). All G1407 isolates that required increased MICs of ceftriaxone or cefixime had *penA* allele XXXIV. Among the 718 isolates in this study, none had reduced susceptibility to azithromycin and an ESC. The relatively high prevalence of this strain type, combined with the evolutionary capacity of *N*. *gonorrhoeae* to evade antimicrobial drug pressure, may eventually lead to circumstances where even dual therapy is not sufficient.
